# Effects of lumbar-pelvic training combined with electroacupuncture on chronic nonspecific low back pain

**DOI:** 10.1097/MD.0000000000034407

**Published:** 2023-07-21

**Authors:** Yuandong Cheng, Yingli Yu, Yuqin Wang, Ao Fan, Hua Yang, Hailiang Wang, Liugang Tang

**Affiliations:** a Sichuan Province Orthopedic Hospital, Chengdu, China; b Sichuan Electric Power Hospital, Chengdu, China; c Meishan Traditional Chinese Medicine Hospital, Meishan, China.

**Keywords:** clinical efficacy, electroacupuncture, lumbar-pelvic training, nonspecific low back pain

## Abstract

This observational study was conducted to investigate the effect of lumbar-pelvic training (LP) combined with electroacupuncture (EA) in the treatment of chronic nonspecific low back pain. One hundred and twenty patients diagnosed with chronic nonspecific low back pain were evenly randomized to receive the following 4 treatments for 2 weeks: LP combined with EA (Group A), EA (Group B), LP (Group C) or no intervention (Group D). The LP was a self-developed training program containing 5 movements and was conducted three times a week to build up the strength of abdomen muscle groups. Four acupoints along the foot-*taiyang* bladder meridian and the governor vessel were chosen for EA five times a week based on the theory of Traditional Chinese Medicine. The Visual Analog Scale and Oswestry Disability Index were measured before and after treatment to assess the reduction of pain intensity and functional disability, respectively. Following the treatments, Visual Analog Scale and Oswestry Disability Index scores in all 3 intervention groups were significantly lower than those in the Group D without intervention (*P* < .01). Among the intervention groups, Group A’s scores were lower than those of Group B or Group C (*P* < .01). The overall efficacy of Group A was 93.33%, which was higher than that of Group B (76.67%) and Group C (70.00%) (*P* < .01). In conclusion, this study suggest that our self-developed lumbar-pelvic training combined with electroacupuncture is effective for chronic nonspecific low back pain in terms of pain and disability reduction.

## 1. Introduction

Nonspecific low back pain (NSLBP) is a major kind of low back pain (LBP) defined as pain localized below the margin of the last ribs and above the inferior gluteal lines without identifiable, known specific pathological changes.^[1,2]^ LBP has become the leading cause of disability globally, among which NSLBP accounted for more than 85% of LBP.^[3,4]^ It is reported that the mean lifetime prevalence of LBP and chronic LBP were up to 38.9% and 20.1%, respectively.^[5]^ Nearly 540 million people of all age suffered from activity-limiting LBP at some point globally, with a peak prevalence in people between 40 and 69 years.^[3,5]^ However, the prevalence of LBP increased steeply during teenagers in recent years.^[6]^ As population increase and aging, the disability duration of LBP increased by 54% between 1990 and 2015.^[7]^ The global burden of NSLBP would continue to rise in the coming decades.

Due to its refractory and recurrent nature, NSLBP is an extremely frustrating symptom that affects a large number of people.^[8]^ Based on modern medical studies, the possible triggers of NSLBP could be attributed to instability of the lumbar vertebrae caused by loss of abdominal core strength,^[9]^ degeneration of lumbar facet joint,^[10,11]^ insufficient perfusion in local capillary with toxics deposition^[12]^ or immune disorder.^[11]^ NSLBP lacked disease-modifying therapy due to the unascertained etiology. The current rehabilitation therapy focuses on relieving pain, strengthening the trunk muscles, and improving the motor function of lower back, thus preventing functional defect and work disability.^[13]^ Published guidelines from different countries has proposed self-management, education, pharmacological agents, and non-pharmacological approaches as treatments for NSLBP. Non-pharmacological therapies, such as exercise and acupuncture, were particularly recommended for chronic NSLBP.^[14]^ However, the most effective interventions for NSLBP are still under discussion.

Exercise is commonly used in the management of LBP. Guideline in UK advised people with LBP that staying physically active is likely to be beneficial.^[15]^ Various core stability exercises were adopted for LBP treatment to restore or augment the control and support of the spine and pelvis, which showed significant reduction in pain intensity and functional disability, accompanied by the activation of deep trunk muscles.^[16–18]^ A study found that exercise could reduce oxidative stress damage and improve the levels of functional molecular components in the intervertebral disc, thus alleviating degeneration of lumbar disc.^[19]^ Nevertheless, most literature provided little and ambiguous guidance for patients to determine the exact way of exercise implement.

Acupuncture is an ancient Chinese method to relieve pain. Nowadays, it has been widely used in the treatment of NSLBP in many countries, and has been recommended in guidelines from the US, UK and Canada.^[20,21]^ In Traditional Chinese Medicine, LBP was thought to be the manifestation of kidney deficiency, for which the corresponding treatment focused on maintaining kidney function. Acupoints on the foot-taiyang bladder meridian, which located along the lumbosacral and lower limb, were chosen to treat LBP, since kidney and bladder are 2 organs complementing each other. Electroacupuncture (EA) served as a modified variation of traditional acupuncture, with electricity stimulation to enhance the benefits of traditional therapeutic method. Studies have showed that EA could improve the Visual Analog Scale (VAS) and Oswestry Disability Index (ODI) score of NSLBP patients, implying an effective way for pain reduction.^[22,23]^

Consequently, previous studies have shown that exercise and EA are effective in the treatment of NSLBP. However, there is little evidence to support the use of one intervention combined with another. In this study, we developed an exercise program of lumbar-pelvic training (LP) based on our clinical experience, and aimed to compare the effects of LP combined with EA, LP alone, EA alone for chronic NSLBP patients on the reduction of pain intensity and functional disability.

## 2. Methods and patients

### 2.1. Study design and setting

This study was conducted at the following 3 hospitals in Sichuan, China: Sichuan Province Orthopedic Hospital, Meishan Traditional Chinese Medicine Hospital, and Sichuan Electric Power Hospital. All subjects gave their informed consent before they participated in the study. The study was conducted in accordance with the Helsinki Declaration for human studies and was approved by the Ethics Committee of Sichuan Province Orthopedic Hospital (KY2020-037-01). Only data from eligible patients were used to minimize bias in this research.

### 2.2. Participants and study size

**Figure 1.**
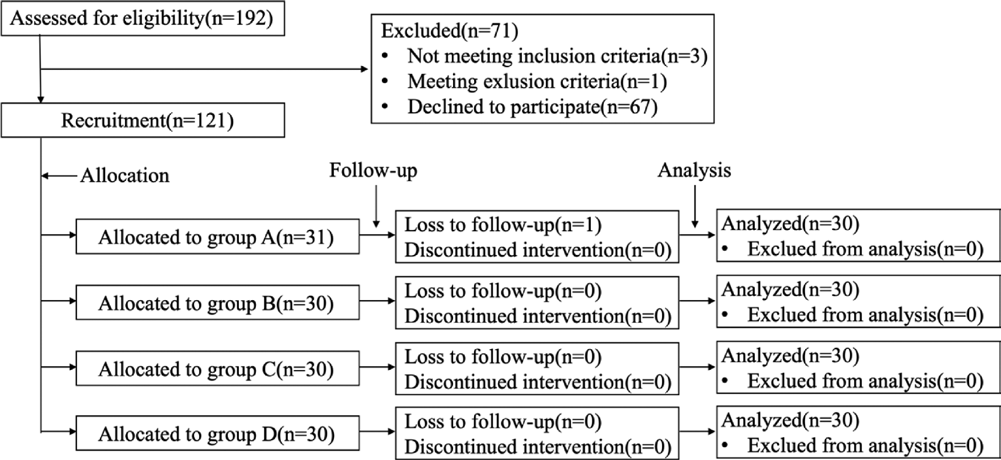
Flow diagram of participants.

In this study, we enrolled patients diagnosed with chronic NSLBP from April 2020 to April 2021. Our sample size calculation was based on the detection of a 1.5-point difference in VAS score post treatment between the single and combined interventions. We estimated the mean and standard deviation of the single intervention group from a previously published study^[24]^ (EA) and our pilot study (LP). Using an alpha level of 0.05 and 90% power, we determined that a minimum of 27 participants was needed per group. To account for possible dropouts, we ultimately assessed 192 patients for eligibility (Fig. 1). Of these, 71 were excluded for noncompliance with the inclusion criteria (n = 3), exclusion criteria (n = 1), and unwillingness to participate in the study (n = 67). The rest 121 participants were randomized into 4 groups: lumbar-pelvic training combined with electroacupuncture (Group A, n = 31), electroacupuncture (Group B, n = 30), lumbar-pelvic training (Group C, n = 30) or no intervention (Group D, n = 30). One patient in group A was unable to keep up with the follow-ups. Therefore, data from a total number of 120 participants were analyzed in the results. The information of these 120 patients in each group is shown in Table 1. There are no significant differences in sex ratio, age, and disease duration among groups.

**Table 1:** Characteristics of subjects in each group.

Group	Sex ratio (male/female)	Age (mean ± SD)	Disease duration (months, mean ± SD)
A (LP + EA)	16/14	38.73 ± 10.39	22.40 ± 8.21
B (EA)	14/16	40.17 ± 10.67	21.07 ± 9.04
C (LP)	18/12	39.96 ± 10.42	20.93 ± 9.12
D (control)	15/15	41.23 ± 11.04	21.70 ± 8.72
Test statistic	χ^2^ = 1.170	*F* = 0.2790	*F* = 0.1761
*P* value	.76	.84	.91

EA = electroacupuncture, LP = lumbar-pelvic training, SD = standard deviation.

### 2.3. Eligible criteria

According to Criteria of Diagnosis and Therapeutic Effect of Diseases and Syndromes in Traditional Chinese Medicine,^[25,26]^ chronic NSLBP was diagnosed as follows: experience of long-term sickness (more than 12 weeks), lingering dull pain, or soreness in the lower back from the 12th rib to the gluteus maximus area; recurrent or progressively chronic exacerbation without nerve root involvement; and exclusion of any other internal medical complications, severe osteoporosis, lumbar disc protrusion, or lumbosacral stenosis.

Inclusion criteria were as follows: age between 20 and 60 years (inclusive); in line with the above-mentioned diagnostic criteria for chronic NSLBP; and ability to comply with the study protocol and provide informed consent.

Exclusion criteria were as follows: previous spinal cord injury or surgery; suffering from ankylosing spondylitis, lumbar fissures, lumbar disc herniations, or lumbar spondylolisthesis; being pregnant or nursing; or suffering from bone tuberculosis, tumors or other lesions caused by medical system diseases.

### 2.4. Drop-out criteria

Drop-out criteria are as follows: refusing further participation in the study; being unable to proceed due to adverse reactions, unexpected events, or deteriorating conditions; or not keeping up with follow-ups.

### 2.5. Lumbar-pelvic training

**Figure 2.**
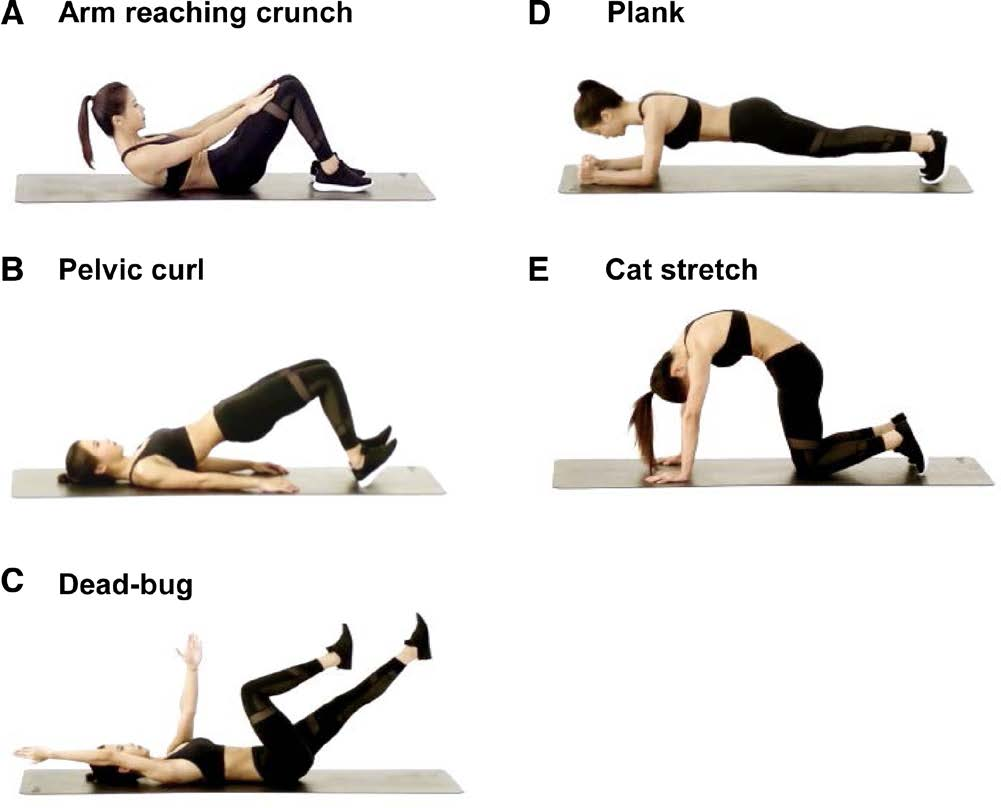
Instructions for each movement in lumbar-pelvic training. (A) Arm reaching crunch. (B) Pelvic curl. (C) Dead-bug. (D) Plank. (E) Cat stretch.

Based on clinical practice, we developed lumber-pelvic training. One session of lumbar-pelvic training consists of 5 movements: arm reaching crunch, pelvic curl, dead-bug, plank, and cat stretch (Fig. 2). The plank was performed once and the other 4 movements were repeated fifteen times in turn in 1 session. Each session was repeated 3 times at 1-minute intervals. The training was performed 3 times a week for 2 weeks. Detailed training protocols are as follows:

#### 2.5.1. Arm reaching crunch

Start by lying in a supine position with your knees bent, your feet flat on the ground, and your legs a fist-distance apart. Put both hands on your lap and extend them straight forward. By inhaling deeply, tighten the abdominal muscles to propel the shoulders and upper back upward sequentially until both hands touch the knees. Exhale as you return. As you move through the process, it’s crucial to keep the chin close to the neck, your lower back grounded, and your arms straight. This will maintain steady control of the abdominals throughout.

#### 2.5.2. Pelvic curl

Lie flat on your back with your knees bent at a 90° angle, your legs roughly shoulder distance apart, your arms relaxed at your sides with your palms down, and your pelvis in a neutral position. Inhale to stabilize your abdominals and tighten your hips. During exhalation, tighten the abdominal muscles and lift the pelvis and spine off the floor. As a result, the knees, hips, and shoulders will be straight. As you inhale again, lower your spine until your tailbone touches the ground. While performing this movement, keep in mind that the head and shoulders are always attached to the ground.

#### 2.5.3. Dead-bug

Lie supine on your back with your knees and hips bent. Lift the feet off the ground until the upper legs are vertical. Extend your arms so that they are vertical to the ground. Slightly elevate the hips off the ground, keeping the upper back close to the ground. During an inhalation, extend 1 leg forward and raise the opposite arm overhead until they are almost parallel to the ground. Then exhale and return to the starting position. Follow the same procedure with the other leg and arm. It is important to note that the back stays on the ground throughout the whole procedure, while the legs remain elevated.

#### 2.5.4. Plank

Start in a pushup position and place the elbows flat on the ground at shoulder distance apart. Push the ball of your foot on the ground while keeping your arms vertical to the ground. Ensure your body is in a straight line by tightening your abdominal muscles and lifting your hips. Be sure to keep your spine in a neutral position and breathe evenly. Patients who are unable to do this can use their knees to support their body. Stay in the position until you are completely exhausted. Throughout this process, the abdominal and gluteal muscles must always be tightened to prevent the waist and back from falling.

#### 2.5.5. Cat stretch

The first step is to begin with an all-fours position with the back straight and flat. As you exhale, rotate your pelvis forward slowly to curve each vertebra one at a time, hold your chin downward, and hunch your spine upward. Inhale to return to the starting position. Breathe in rhythm with the movement of your pelvis and vertebra.

### 2.6. Electroacupuncture

Based on the guideline of acupuncture for LBP and literature suggestion,^[26,27]^ the following 4 acupoints were adopted in this study: Shenshu, Dachangshu, Guanyuanshu, and Zhibian (Table 2). These 4 points are located along the foot-taiyang meridian and the governor vessel, the stimulation of which could eliminate blockage in this meridian, thus relieving lumbar muscular spasm and pain. EA was conducted 5 times a week for 2 weeks. Briefly, the filiform needles (Huatuo, 0.30 mm × 75 mm) were inserted towards the spine at 15° to the derma. The needle was lifted slightly with the tip lateral against the derma when arriving at the facet joint. This was done by pushing the needle slightly along the facet joint to the ventral area to feel the abnormal sensation. The needles were connected to an electroacupuncture apparatus (G6805-II) and the stimulation lasted for 15 to 20 minutes at a frequency of 2 Hz.

**Table 2:** Acupoints used in the treatment.

Acupoints	Location	Major indication
Shenshu BL23	1.5 inches lateral to the lower border of the spinous process of the 2nd lumbar vertebra	Disorders in the urinary system, diseases in the genital system, tinnitus, deafness, chronic lumbago
Dachangshu BL25	1.5 inches lateral to the lower border of the spinous process of the 4th lumbar vertebra	Large intestinal diseases, lumbar muscle strain, or sprain
Guanyuanshu BL26	1.5 inches lateral to the lower border of the spinous process of the 5th lumber vertebra	Abdominal distension, diarrhea, lower back pain, frequent urination, or retention of urine
Zhibian BL54	3 inches lateral to the lower border of the spinous process of the 4th sacral vertebra	Pain in the lumbosacral area, dysuria, constipation, hemorrhoids, vulvitis

### 2.7. Outcome evaluation

The outcomes were evaluated before and after the 2-week treatment. The outcome measures included pain intensity (measured by VAS) and disability severity (evaluated with ODI). In the VAS assessment, pain intensity was measured using a ten-centimeter line.^[28]^ In the ODI measure, the patients were asked to complete questions from ten sections and then scored on a scale of 1 to 100, with scores 1 to 20 indicating minimal disability for which no treatment is needed and scores 21 to 40 indicating moderate disability which can be managed by conservative means.^[29,30]^

Based on Criteria of Diagnosis and Therapeutic Effect of Diseases and Syndromes in Traditional Chinese Medicine,^[25]^ the efficacy was defined as recovery: the clinical symptoms and signs almost disappear with the ability to engage in work and household activities; improvement: the clinical symptoms and signs are relieved with the ability to engage in most work and household activities; or inefficient: the clinical symptoms and signs remain unchanged.

### 2.8. Statistical analysis

The data were analyzed by Statistical Package for Social Sciences 22.0 (SPSS 22.0; IBM, Armonk, NY) and presented as mean ± standard deviation (SD). The sex ratio of participants was assessed by chi-square test. The mean differences among multiple groups were calculated by one-way ANOVA. For samples with equal SDs, Turkey’s post hoc test was used for multiple comparisons, and for samples with different SDs, Dunnett’s T3 post hoc test was used. Therapeutic effects were analyzed by Kruskal–Wallis test by ranks and one-way ANOVA followed by Student-Newman-Keuls post hoc test.

## 3. Results

### 3.1. VAS scores

The participants were tested with VAS before and after the 2-week treatment. Each group’s pain intensity is homogeneous before therapy (*P* = .73). Compared with group D, group A, B, and C’s scores were significantly lower after treatment (A vs D: *P* < .001; B vs D: *P* < .001; C vs D: *P* < .001). Additionally, we found that the combination therapy score was lower than those in single therapy (A vs B: *P* < .001; A vs C: *P* < .001), while there were no significant differences between group B and C (B vs C: *P* = .93). Together, these data indicate that LP is effective regarding pain relief for chronic NSLBP patients, and its combination with EA could provide more benefits than single therapy (Table 3).

**Table 3:** VAS scores (mean ± SD, points).

Group	n	Baseline	Post treatment
A (LP + EA)	30	7.13 ± 1.12	1.63 ± 0.93*
B (EA)	30	6.93 ± 1.11	2.90 ± 1.24*,†
C (LP)	30	7.13 ± 1.17	3.17 ± 1.02*,†
D (control)	30	6.87 ± 1.11	4.87 ± 1.05

EA = electroacupuncture, LP = lumbar-pelvic training, SD = standard deviation, VAS = Visual Analog Scale.

* *P* < .01 compared with group D.

† *P* < .01 compared with group A.

### 3.2. ODI scores

The ODI questionnaire was used to measure the patients’ functional disability. Consistent with VAS scores, the patients had similar baseline ODI scores (*P* = .93), which indicated moderate disability in clinical practice (ODI scores 21–40). After the 2-week intervention, the ODI scores in group A, B, and C were significantly lower than that in group D (A vs D: *P* < .001; B vs D: *P* < .001; C vs D: *P* < .001), and fell into the range of minimal disability (ODI scores 0–20). Moreover, the score in group A was also significantly lower than those in group B and C (A vs B: *P* < .001; A vs C: *P* < .001), but the effects in group B and C were similar (B vs C: *P* = .93) (Table 4).

**Table 4:** ODI scores (mean ± SD, points).

Group	n	Baseline	Post treatment
A (LP + EA)	30	30.83 ± 6.58	6.40 ± 4.08*
B (EA)	30	30.71 ± 7.52	12.47 ± 3.12*,†
C (LP)	30	31.77 ± 6.65	13.27 ± 3.73*,†
D (control)	30	31.03 ± 6.66	25.97 ± 6.76

EA = electroacupuncture, LP = lumbar-pelvic training, ODI = Oswestry Disability Index, SD = standard deviation.

* *P* < .01 compared with group D.

† *P* < .01 compared with group A.

### 3.3. Overall efficacy

After intervention, the participants were categorized into 3 groups: Recovery, Improvement, and Inefficient. Kruskal–Wallis test and Student–Newman–Keuls post hoc test showed that the overall efficacy in group A was significantly different from that in group B, C, and D; group D was different from group B and C; but group B and C were not statistically different (*P* < .001) (Table 5).

**Table 5:** Overall efficacy [cases (%)].

Group	n	Recovery	Improvement	Inefficient	Overall efficacy
A (LP + EA)	30	18 (60.00)	10 (33.33)	2 (6.70)	28 (93.33)
B (EA)	30	10 (33.33)	13 (43.33)	7 (23.33)	23 (76.67)
C (LP)	30	9 (30.00)	12 (40.00)	9 (30.00)	21(70.00)
D (control)	30	0 (0)	0 (0)	30 (100)	0 (0)

EA = electroacupuncture, LP = lumbar-pelvic training.

## 4. Discussion

Exercise is recommended to be a part of low back pain management in several clinical guidelines, and some exercise approaches require a long time (for instance, thirteen weeks) to be effective.^[31–34]^ This is partially due to the fact that different exercise intensities and methods are used within different exercise protocols. In the present study, we developed a novel 2-week home exercise protocol named lumbar-pelvic training. After that, we tested the therapeutic effects of LP and LP combined with EA. The data showed that LP was as effective as EA in reducing pain and disability in the short term. Unlike EA, which requires equipment and clinical experience that are not always readily available, LP is a cost-effective and time-saving intervention that can be performed at home. In light of this, LP could be a potential therapy for managing LBP.

Combination therapies, such as exercise combined with other methods, are also used to treat LBP.^[35–38]^ In the present study, we evaluated the effect of LP in combination with EA. The VAS and ODI scores are significantly lower in the combination group than in the single therapy groups. Furthermore, combined therapy (93.33%) has a higher overall efficiency than LP (70.00%) or EA (76.67%). In summary, LP alone and LP combined with EA are both effective for managing chronic LBP with regard to pain relief and disability reduction.

Our research on the effect of EA is consistent with earlier studies.^[24,39]^ Despite the thousands of years that acupuncture has been practiced to treat LBP, a lack of solid mechanistic evidence has restricted its use in the modern era. Early studies reported that somatosensory autonomic reflexes are regulated by channels during EA.^[40]^ Research on mice recently discovered 2 reflexes (vagal-adrenal reflexes and spinal-sympathetic reflexes) that are driven by EA.^[41,42]^ Following these discoveries, a candidate pathway was discovered, involving prokineticin receptor 2 positive sensory neurons, the dorsal motor nucleus of the vagus, and adrenal glands secreting catecholamines to inhibit inflammatory responses.^[43]^ These data fill a gap in EA research and could provide neuroanatomical support for EA practice.

There are some limitations in this study. Based on disease duration, LBP patients are usually categorized into acute (less than 6 weeks), subacute (6–12 weeks), and chronic groups (more than 12 weeks).^[44]^ This study included only chronic LBP patients. Further evaluation of the effect of LP alone and in combination with EA could be conducted in acute and subacute LBP patients. Previous studies suggest that it is also possible to prevent LBP with exercise.^[34,38,45–47]^ It is currently unknown whether our self-developed LP and its combination with EA could reduce or even prevent relapses of LBP. More in-depth research is needed to address these questions to provide comprehensive evidence for clinical practice.

## 5. Conclusion

Our self-developed lumbar-pelvic training could serve as a promising treatment for chronic nonspecific low back pain in terms of reducing pain and disability. Its combination with electroacupuncture could provide better clinical outcomes than either treatment alone.

## Author contributions

**Conceptualization:** Yuandong Cheng, Liugang Tang.

**Data curation:** Yuandong Cheng, Ao Fan.

**Formal analysis:** Yuandong Cheng, Yuqin Wang, Hua Yang.

**Funding acquisition:** Yuandong Cheng, Liugang Tang.

**Investigation:** Ao Fan, Hailiang Wang.

**Methodology:** Yuandong Cheng, Yingli Yu, Yuqin Wang, Ao Fan, Hailiang Wang.

**Project administration:** Liugang Tang.

**Resources:** Yuandong Cheng, Ao Fan, Hailiang Wang, Liugang Tang.

**Software:** Yuandong Cheng.

**Supervision:** Liugang Tang.

**Validation:** Yingli Yu, Yuqin Wang, Hua Yang.

**Visualization:** Yingli Yu, Yuqin Wang, Hua Yang.

**Writing – original draft:** Yuandong Cheng, Liugang Tang.

**Writing – review & editing:** Yuandong Cheng, Liugang Tang.
